# The new frontiers of learning in otorhinolaryngology: a reflection about challenges and advances

**DOI:** 10.1016/j.bjorl.2023.101370

**Published:** 2023-11-28

**Authors:** Sofia Oliva-Costa

**Affiliations:** Otolaryngology Department of State University of Campinas (UNICAMP), Rua Tessália Viera de Camargo, 126, CEP 13083-887 Campinas, SP, Brazil

Otorhinolaryngology is a specialty that requires multiple skills in its practice: clinical reasoning, handling of diagnostic tools, interpretation of specific exams, outpatient procedures skills and surgical skills. Works in different scenarios: operating room, emergency room, infirmary, intensive care unit, and outpatient clinic. Provide care to patients of all age groups and genders. In addition to this wide range of skills needed, diverse workplaces, and heterogeneous populations, it also has several fields of interest. In addition to the primary ones that give the specialty its name, many others have emerged with the advancement of science. Given this scenario, arises the discussion about the adequacy of learning models in otorhinolaryngology residency to the current moment.

According to the last census published by ABORL-CCF in 2018, Brazil had 8644 otorhinolaryngologists, representing one specialist for every 24,024 inhabitants. It had 113 registered residency programs.^1^ It remains one of the countries with the shortest duration of the otorhinolaryngology residency program, with just three years, while training is usually more extended in other countries, varying between 4–6 years in European countries and the United States. The reduced time to acquire the same skills and knowledge could be the driving force behind a trend observed in the country: pursuing a fellowship for subspecialization. It will provide a similar duration of training compared to other countries but leaves room for questioning about the quality of general otorhinolaryngologists’ training.

There has been a movement among surgical specialties to abandon the traditional time-based learning model and embrace the competency-based model. In the conventional time-based model, trainees finish their training after completing the established period under the tutelage of a team of specialists with expertise in the area, also known as the “apprentice and master model,” introduced by Halsted and Osler at the end of the century XIX. However, in this model, the trainee is not guaranteed to acquire the necessary competency during this period. Unusual situations can negatively interfere with this training process, such as temporary technical limitations of the service or public health situations such as the global COVID-19 pandemic. In the competency-based model, the trainee progresses in the program when they acquire a competency. The barrier to the evolution of this model has been determining the best tool to attest to a competence acquisition.^2^

New patient safety initiatives and pressure for cost efficiency in operating rooms have limited opportunities for developing technical skills. Even teaching using cadaver parts, the gold standard in teaching surgical skills outside patient care, has been limited due to ethical discussions and donor reduction. In this way, alternative ways of teaching skills in surgical specialties outside the patient care have emerged.^3^

Technological advances are helping to resolve the dilemma of balancing patient safety and developing technical skills in training new specialists. The simulation model of learning is becoming popular among surgical specialties as new tools are turning closer to reality, such as virtual reality glasses, surgical consoles, and 3D printing models. This model expands the horizons of teaching and learning technical skills and promises to make the assessment of competence acquisition more objective and reproducible. Some barriers for developing this model are particular to our specialty, once the head and neck is a region of the human body that have complex structures which are difficult to reproduce in a model, and our surgeries require specific instruments, light, and lenses for magnification of surgical field.^3^, ^4^

There is still a lot to be developed in the teaching scenario in otorhinolaryngology. New knowledge, techniques, medications, and instruments will always be emerging. Therefore, one of the most important skills we can acquire in our training process is the ability to adapt to new realities, always driven by the desire to keep learning.

## Conflicts of interest

The author declares no conflicts of interest.
